# Faces of time: a historical overview of rapid innovations in coding animal facial signals

**DOI:** 10.3389/fvets.2025.1716633

**Published:** 2025-11-10

**Authors:** Teddy Lazebnik, Brittany Florkiewicz

**Affiliations:** 1 Department of Information Systems, University of Haifa, Haifa, Israel; 2 Department of Computing, Jönköping University, Jönköping, Sweden; 3 Department of Psychology, Lyon College, Batesville, AR, United States; 4 Research Center for Human-Animal Interaction, College of Veterinary Medicine, University of Missouri, Columbia, MO, United States

**Keywords:** facial signals, facial action coding systems, artificial intelligence, animal communication, facial expression

## Abstract

Since Charles Darwin's influential work on *The Expression of the Emotions in Man and Animals*, there have been significant advancements in how animal behaviorists identify and describe the facial signals of animals, including humans. Most of these advancements are largely attributed to technological innovations in how data are recorded in addition to the establishment of computer programs that aid with behavioral coding and analysis. Consequently, various manual and automated approaches can be adopted, each with its own benefits and drawbacks. The goal of this overview is twofold. First, we provide an overview of the past and present techniques for coding animal facial signals. Second, we compare and contrast each method, offering multiple examples of how each technique has been used and can be applied in the study of animal facial signaling today. Our examples include studies that address empirical questions related to animal behavior, as well as studies aimed at generating applications for animal welfare. Instead of favoring or criticizing one approach over another, our aim is to foster appreciation for the advancements in animal facial signal coding and to inspire future innovations in this field.

## Animal facial signals

For decades, researchers have studied animal facial signals, focusing on their physical forms, social functions, and emotional links (1–3). These facial signals are generally defined as combinations of one or more facial muscle movements that animals produce during bouts of communication (4–6). Notably, these combinations of movements often seem unrelated to basic biological functions, such as chewing (7–9), and can be directed toward other animals (10). Some researchers suggest that facial signals in animals are closely linked to specific categories of emotional arousal, providing valuable non-invasive insights into their mental lives (1, 11, 12). However, these facial signals, along with the corresponding muscle movements, are subject to interpretation by others (13, 14). Consequently, some researchers propose that these signals can reliably predict future behaviors, allowing individuals to adapt their own actions based on the perceived meaning of these signals (3, 13–16). The study of facial signals in non-human animals is particularly important for humans, as we live alongside a variety of species, both in wild and captive settings. By understanding the social functions and emotional links associated with specific combinations of facial muscle movements, we can adjust our behavior, which in turn improves our interactions with non-human animals. For example, understanding the facial muscle movements linked to a higher likelihood of aggression during intraspecific interactions among domesticated cats enables us to intervene before conflicts occur (17). In the context of cat-human interactions, this knowledge allows us to adjust our own behavior to minimize the risk of injury (18).

## Manual coding approaches

The study of animal facial signals has been greatly influenced by technological innovations available to researchers (19). For example, early naturalists heavily relied on illustrations and written descriptions (12, 20, 21) until the advent of photography and videography. Over the past decade, researchers have made significant progress in identifying and differentiating various types of facial signals (3, 22). Some of the earliest methods involved creating ethograms, which categorize facial signals based on key similarities in facial muscle movements, while also considering the social context of the interaction (12, 23). In his influential work, *The Expression of the Emotions in Man and Animals*, Charles Darwin differentiates between six facial signals type associated with distinct categories of emotion (12, 24). Through behavioral observations, photographs, illustrations, and conversations with fellow naturalists, Darwin established these categories to highlight similarities in physical forms and emotional responses among human and non-human animals. Darwin documented not only the movements of facial muscles but also various social factors influencing their production, including the presence or absence of other animals and the perceived bond between them, such as the relationship between humans and domesticated animals (12).

### Facial action coding systems (FACS)

Almost a decade after Darwin's work on animal facial signals, researchers and practitioners began to develop more systematic and standardized methods for studying these signals. One of the early pioneers was Carl-Herman Hjortsjö, who focused on facial mimicry and the silent communication conveyed through individual facial muscle movements in humans (5). He suggested that these movements are similar to letters in human language, where combinations form “words” that can be easily understood due to their connections with different emotional states (5). To investigate this “silent language,” Hjortsjö created a coding system that assigned numerical codes to specific facial movements in humans, allowing for the identification of unique facial muscle movement combinations (or signals). For example, while both “suspicious” and “observing” facial signals share lowered brows, they differ from one another based on chin position (5).

Following Hjortsjö's work, Paul Ekman developed the human Facial Action Coding System (FACS) in 1978. He argued that the “language” of emotional facial signals was “universal” across human societies, and that the meaning of each signal could be identified based on individual muscle movements (11). During his research, Ekman noted that certain cultures had “display rules” or socially constructed facial signals, making it difficult to identify the “true” underlying emotion being experienced (25). To understand the true meaning (i.e., emotional link) of these facial signals, Ekman developed the FACS (6, 26). FACS employs both posed photographs and video to train individuals in recognizing subtle and overt facial muscle movements (called Action Units, or AUs) that are combined to create a signal (6, 26). Facial signals can be captured and coded through photographs, video clips, or in real time. Together, Hjortsjö and Ekman's coding systems represent the most comprehensive and systematic approaches to studying human facial signals, minimizing observation bias by considering all facial movements equally (27). It is important to recognize that both Hjortsö's and Ekman's coding schemes focus on the physical aspects of human facial signals (22). Researchers using these protocols are tasked with creating additional metrics and measures that link these physical forms to their socio-emotional functions.

Recently, efforts to systematize and standardize the study of non-human animal facial signals have led to the development of FACS for various animal species, collectively known as animalFACS [https://animalfacs.com/; (22)]. These systems have been developed for a diverse range of species, including non-human primates (27–35) and domesticated animals (36–38). As a result, our understanding of animal facial signals has significantly improved. Recent studies indicate that some mammals can produce dozens of distinct facial muscle movements for communication, surpassing what was previously documented (9, 17, 39, 40). Researchers utilizing animalFACS have found that specific combinations of facial muscle movements correlate with distinct social outcomes, highlighting the direct relationship between the physical form of facial signals and their social functions (41, 42). Furthermore, studies indicate that animals' facial signaling behaviors are influenced by multiple factors such as the strength of social bonds (39), levels of social tolerance (43), and group size (40), with noticeable variations both within and between species (40). AnimalFACS facilitates cross-species comparisons by providing a consistent method for identifying and documenting facial muscle movements, thereby ensuring more accurate assessments across different species (22).

Finally, FACS are now used to assess animal welfare by identifying key facial muscle movements linked to pain and other negative emotions (44). For instance, in domesticated animals, facial muscle movements described in FACS such as AU143 (blink), AU145 (eye closure), and lip corner puller (AU12) has been observed in facial behaviors associated with pain among domesticated cats (45) and dogs (46). It is important to note that in studies of pain among non-human animals without an established FACS, such as rodents, researchers often employ similar systematic and standardized methods for identifying facial muscle movements, taking inspiration from FACS-based approaches (47). In these cases, researchers identify, describe, and examine Facial Action Units (FAUs), specific facial movements that occur during pain episodes (46, 48), highlighting the significance and practicality of FACS-based approaches.

### Limitations of FACS coding

**Figure 1:**
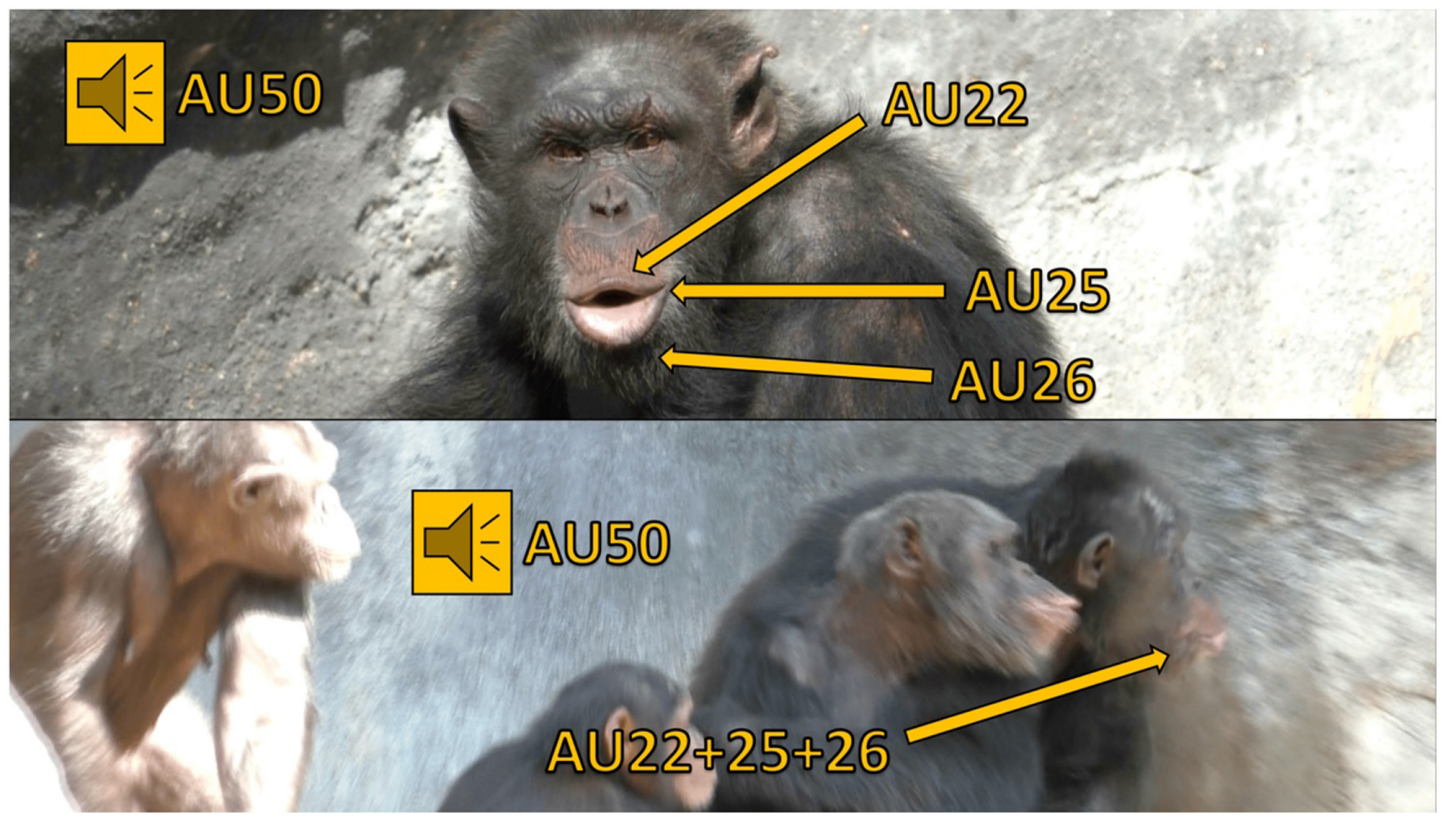
This figure features a chimpanzee named Ben producing pant hoots, identifiable by the presence of AUs 22+25+26+50 (i.e., the lips are funneled, lips parted, and jaw dropped, all accompanied by a vocalization). Pant hoot calls are generated during periods of high excitement, such as responses to observing playful interactions, distress, or the availability of food (51). In this scenario, Ben produces a pant hoot facial signal and vocalizes in response to another chimpanzee's scream, causing the surrounding chimpanzees to heighten their vigilance and attend to the situation. In the top image, Ben's face is clearly visible as he sits still, devoid of any other chimpanzees or objects that could obscure his facial muscle movements. Conversely, the bottom image presents challenges for FACS coding: Ben's face (on the right) is partially obscured by other chimpanzees, and he is in motion, moving quickly from left to right. Additionally, the profile view in the video clip complicates the discernment of his facial muscle movements compared to a head-on perspective.

Although animalFACS are systematic and standardized methods that have enhanced our understanding of animal facial signals, they are associated with multiple drawbacks. Many of these limitations stem from the manual coding required, as researchers must analyze photographs, videos, or real-time behaviors to identify the presence or absence of many different facial muscle movements. First, to use animalFACS, researchers must first undergo training and pass a certification test that evaluates their reliability in coding facial movements when compared to FACS experts (22, 49). This process is crucial for ensuring consistency in published studies and applications, but it can be quite time- and resource- intensive (49). Second, coding using FACS-based approaches is extremely time-consuming; just a few seconds of video footage can take several hours to analyze (50). Even coding photographs is a lengthy process, as researchers must assess dozens of distinct facial muscle movements. Third, given the number of facial muscle movements documented in animalFACS, type 2 coding errors may occur when attempting to code all possible Action Units (AUs). Finally, coding accuracy and efficiency are largely influenced by many external factors, such as the visibility of subjects and the number of subjects within a given setting (Figure 1).

## The artificial intelligence revolution

By building upon the FACS system, which offers an objective framework for characterizing facial behavior, researchers were able to formalize animal communication in a manner consistent with modern data analysis (52). This data-driven approach, centered on anatomically-based AUs, inherently defines numerical framework such as time series, classification, and regression tasks (48, 53, 54). Despite its promise, the limitations of the FACS systems rendering large-scale, long-duration studies time and resource intensive at best and sometimes even impractical (22, 55, 56). In this context, the advent of artificial intelligence (AI), in general, and computer vision methods, in particular, presented a promising alternative to overcome these limitations (44, 57, 58). By leveraging AI-powered systems, researchers and practitioners aimed to produce objective and rapid scores for facial signals (48, 59, 60). This transition was not a rejection of prior methodologies, but a progression enabled by technology. For example, catFACS have been utilized to develop facial landmark schemes that can automatically detect the presence or absence of specific AUs through video footage (61), allowing for the identification of emotional states such as pain (62). The annotated data painstakingly collected through manual coding became the essential “ground truth” for training data driven algorithms based on novel methods such as machine learning (63, 64). This symbiotic relationship underscores that for any new species to be studied, a foundational, human-led effort to create a species-specific ethogram or coding system must precede the development of an automated solution. The human observer's role has therefore shifted from a primary data collector to a foundational dataset creator and validation expert, a crucial step in the causal chain of modern animal behavior research.

### AI as a tool for pain and welfare assessment

The most impactful early applications of AI in mammalian facial signal coding focused on automating grimace scales, providing a critical tool for assessing pain and welfare, particularly in laboratory and agricultural settings (44). This area of research began with rodents, which are widely used in biomedical studies (65). A pioneering 2018 study detailed the development of an automated Mouse Grimace Scale (aMGS) using a deep convolutional neural network (CNN) architecture (66), specifically a retrained InceptionV3 model (67). This model was trained on a dataset of over 5,700 images and achieved an accuracy of 94% in assessing the presence of pain. The automated scores demonstrated a high correlation with human scores with a Pearson's score of 0.75. By this, demonstrating the machine's ability to accurately replicate and even surpass human-level performance. This principle of grimace scale automation was later extended to other species, including rats, where a study developed an automated Rat Grimace Scale (RGS) that achieved 97% precision and recall for AU detection (65). Similarly, and right after, studies about the usage of AI-based models to automate the Horse Grimace Scale (HGS) were proposed (68–70). These studies employed recurrent neural networks (RNNs) (71) to capture the temporal dynamics of facial signals, a critical factor for accurate pain recognition (70). The results were highly promising, with AI models classifying experimental pain more effectively than human raters.

These studies highlighted the necessity of data augmentation techniques to compensate for the scarcity of annotated horse facial data (20, 21). The success of AI in automating grimace scales fundamentally changes the paradigm of pain assessment. The objectivity, consistency, and ability to analyze vast datasets mean that AI-derived scores can become the new gold standard, potentially more reliable than those from human coders. This transition allows for a shift from reactive, infrequent checks to continuous, proactive monitoring, ultimately leading to improved animal care (72). Across studies reviewed here, automated systems were evaluated against human-coded ground truth derived from certified FACS coders. Typical protocols involved several steps, including: (1) coder training/certification; (2) double-coding subsets and reporting human-to-human reliability (e.g., Cohen's κ/ICC); and (3) using a consensus human label as ground truth for model testing. For Action Unit (AU) detection and facial signal classification, researchers typically reported standard classification metrics such as precision, recall, F1, and accuracy, although these measures are not consistent between studies. Where available, calibration/error metrics (e.g., ROC-AUC, Brier score) and confusion matrices were also reported in some studies. To interpret disagreements between humans and models, multiple studies used third-party adjudication or expert review of discrepant items to determine whether errors arose from annotation ambiguity, image quality/pose/occlusion, or genuine model misclassification.

### Social and emotional states

As the field has matured, AI methodologies have been applied to classify a wider range of emotions and behaviors, enabling new avenues of research in social dynamics and human-animal interaction (73–75). Studies on domestic dogs, for instance, have moved beyond a simple pain/no-pain binary to classify more nuanced emotional states (76–78) developed a method using computer vision and transfer learning with a MobileNet (79) architecture to analyze canine emotional behavior. The model was trained on 1,067 images across four categories: aggressiveness, anxiety, fear, and neutral. While achieving a test accuracy of 69.17%, the research demonstrated the feasibility of using AI to develop tools for dog trainers and handlers, improving the selection and training processes for working dogs. The application of AI to domestic cats addresses a significant gap, as this species is known for its subtle and often enigmatic emotional cues (80, 81) presented a real-time system using convolutional neural networks (CNNs) to classify cat facial signals into four categories: Pleased, Angry, Alarmed, and Calm. The model showed high recognition accuracy and holds substantial potential for applications in veterinary care and enhancing pet-owner communication. Complementing this, (82) leveraged a more specialized architecture, DenseNet (83), which uses dense connectivity patterns to capture intricate features in pet facial signals. This methodological evolution from general CNNs to specialized models like DenseNet and RNNs reflects the growing sophistication of the field and the increasing specificity of research questions.

Beyond welfare, AI is being used in fundamental scientific inquiry to understand the neurobiological basis of facial communication. Studies on primates, such as the work by Chang and Tsao (85), have used computational models to demonstrate how neural ensembles in macaque face patches employ a combinatorial code to represent faces (84, 85). This represents a fascinating reverse application of AI principles, where the study of the brain's own coding mechanisms can inform the development of more efficient AI algorithms (84).

### Limitations of artificial intelligence

While the application of AI to coding animal facial signals has demonstrated immense potential, significant challenges remain. The most persistent obstacle is the scarcity of large, high-quality, and annotated datasets for many species (86, 87). The process of manual ground-truthing, though foundational, remains a bottleneck that limits the development of robust models. Furthermore, models trained on specific breeds or environmental conditions may struggle to generalize to new subjects or different settings (88). The high variability in the facial anatomy across animal species makes a universal, one-size-fits-all model difficult to achieve. Another fundamental challenge lies in the distinction between classifying facial *signals* and interpreting the underlying emotional *state*. While AI is excellent at recognizing and quantifying facial movements, it still cannot fully interpret the internal emotion or intent. The link between a specific facial movement and an internal state (e.g., pain, fear, pleasure) must still be established through careful, human-led behavioral and physiological studies. This human-centric validation remains a critical component of the research process. Several constraints outlined for manual FACS coding also hinder automated performance because they disrupt the facial features models need to detect. Beyond these shared issues, automated approaches introduce additional challenges, including but not limited to: (1) domain shift and dataset bias where models trained on specific facilities, breeds, or camera setups may not generalize without adaptation; (2) annotation noise in the human-provided ground truth used for training; (3) probability calibration/thresholding; and (4) temporal dependence where frame-based models can miss dynamic cues unless explicitly modeled. These considerations motivate reporting human-to-human reliability alongside model-vs.-human agreement, subject-disjoint evaluation, and cross-site tests.

## Discussion

**Figure 2:**
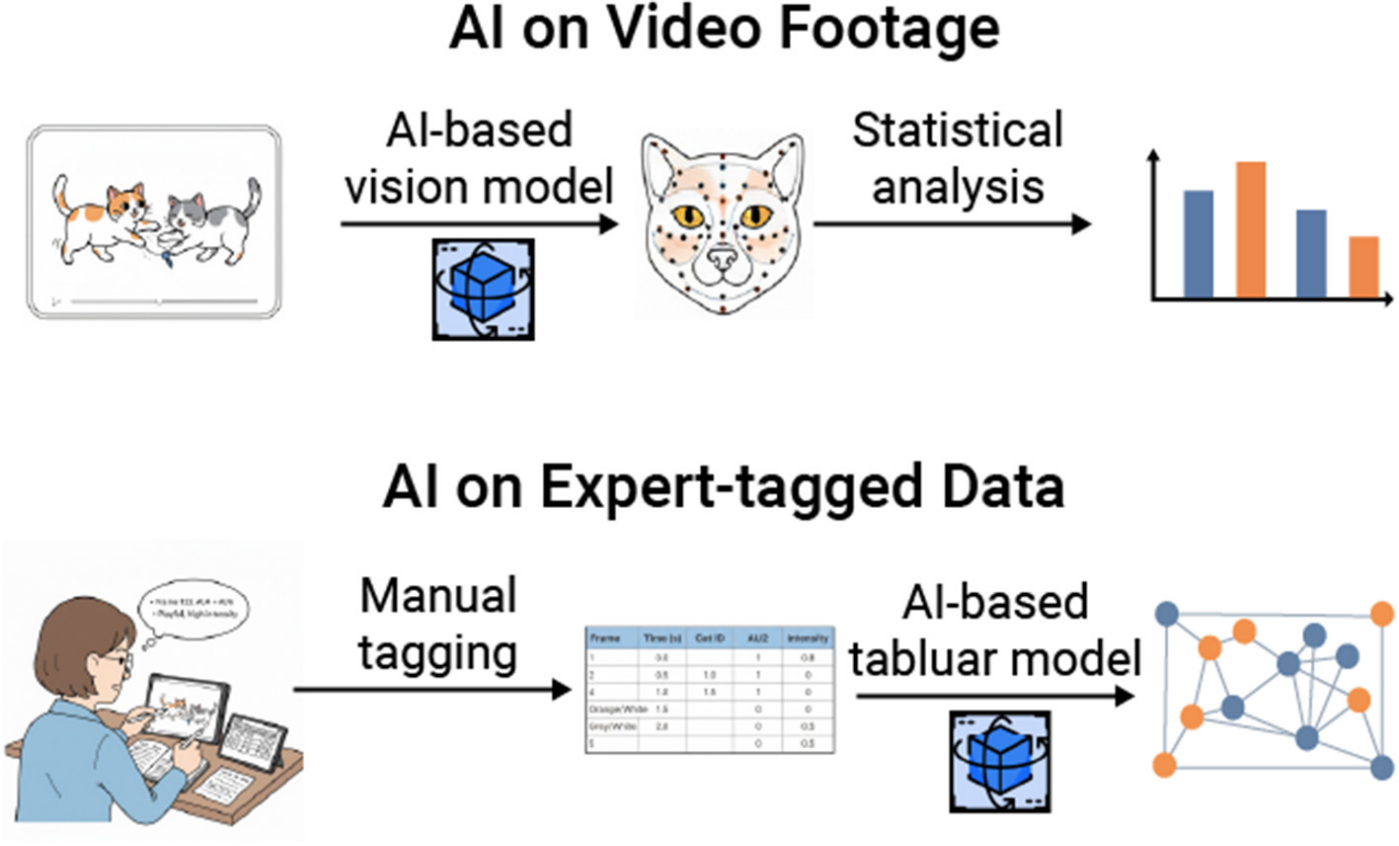
This figure illustrates two primary ways in which both manual and AI-powered approaches can be integrated. The first method involves implementing AI-based vision models on video footage collected by animal behaviorists. These models are used to identify and quantify various variables, such as the distance between animals and the presence/absence of facial muscle movements. The second method involves applying AI-based tabular models to pre-existing datasets that have been manually coded, allowing researchers to explore novel questions. For instance, this may include identifying patterns in communication variables or determining whether instances of rapid facial mimicry are occurring.

Both manual coding and AI-powered approaches offer distinct benefits and drawbacks. To fully leverage the advantages of each method while minimizing their limitations, it is essential to foster collaborative efforts (Figure 2). By combining different kinds of research expertise, more comprehensive studies can also be conducted, bridging gaps in knowledge (89–91). For instance, by utilizing video footage and FACS-coded data, (92) discovered that domesticated cats, like many other mammals, can perform rapid facial mimicry during affiliative interactions. Through the analysis of existing chimpanzee datasets, the authors found that multimodal communication (i.e., where both facial signals and manual gestures are used) along with clear signaling (i.e., which involves employing distinct types of signals during interactions), plays a crucial role in predicting the success of social negotiations among chimpanzees (93). It has been also found that the communicative patterns of chimpanzees vary based on their social rank (94). Understanding how cats communicate can enhance training techniques, inform social interventions, and improve the bond between cats and their owners (95). Similarly, studying chimpanzee communication offers valuable insights into the evolution of human communication and informs conservation efforts, helping to guide decisions about interventions and transfers across accredited institutions. To this end, scholars and practitioners can take advantage of commercial-grade, end-to-end “FaceReader-like” systems for non-human animals. These systems more often than not produce highly accurate results for well-photographed domestic species (e.g., cats, dogs, horses), provided species-specific training data and validated AU/grimace annotations. Early components already exist, such as automated grimace scales and facial-state classifiers for multiple species, on-farm welfare pipelines, and landmark-based cat facial analysis. This demonstrates technical viability and practical utility for this technology and further emphasize that remaining barriers are less about algorithms and more about data coverage (age, breed, morphology), deployment conditions (lighting, camera placement), and standardized validation against certified human coders across sites.

The development of standardized, publicly available datasets for a wider range of species would also accelerate research and improve model generalizability. To this end, benchmarking studies on common tasks in the field and across these emerging datasets can provide researchers and practitioners a quick start from the AI modeling perspective (96–99). Future work may also focus on developing models that can generalize across related species or different taxa, reducing the need to build a new model from scratch for every animal (100–102). As AI becomes more deeply integrated into animal research and care, it is also crucial to consider the ethical implications of using this technology (103, 104), including data privacy and the potential for over-interpretation of animal signals, ensuring that these powerful tools are used responsibly to enhance, not diminish, animal wellbeing. Through large-scale collaborative efforts, such considerations can be addressed more effectively while also advancing our understanding of animal facial signaling.
